# The London Hospital Laundry, Whitechapel, E.

**Published:** 1912-11-16

**Authors:** 


### The London Hospital Laundry, Whitechapel, E.

BY THE LAUNDRY SISTER.

The following account of the London Hospital laundry exhibits the working and cost of a laundry where all the washing is done on the hospital premises.

It has been suggested that a description of a system which makes for efficiency and economy in the management of a hospital laundry would prove useful and interesting to those who are responsible for this important branch of an institution. It would not be possible in a short article to enumerate many details, all more or less important, and perhaps it may be only necessary to touch briefly the principal points.

It must not be supposed that it is intended to criticise the methods of hospital laundries generally, as every institution must, of necessity, "run" the various departments on its own lines; but it ought to be quite possible to organise a system by which efficiency and economy could be established, and also to overcome the difficulties which would no doubt arise in arranging a new system—difficulties which are often considered to be insurmountable, particularly by those who have only been accustomed to one method.

The London Hospital has a fine laundry, which was opened in 1903. The walls are tiled, and easily kept clean, and the system of ventilation is excellent. It is built with entirely separate departments for the staff and hospital washing. The machinery (nine washing machines and other necessary appliances, supplied by Bradford and Co.) is capable of dealing with the whole of the staff and patients' washing, and frequently returns 59,000 to 60,000 pieces weekly. In 1911 the total expenditure on the laundry department was £6,233 16s. 8d. Even this high figure is evidence of economy, in view of the enormous amount of work which was accomplished.

The laundry is entirely under the direction of the matron. In administrative control is a sister-in-charge, who is also a trained nurse, as the matron maintains that trained supervision is necessary in the laundry, as in every other branch of the institution, and this will be readily understood by those who are engaged in institution work. It is not intended to imply that training as a nurse is necessary to secure successful management (though the value of such training could scarcely be over-estimated), but for managing a hospital laundry the training in discipline, punctuality, observation, and a knowledge of the needs of sick people in the wards, would seem to be almost indispensable in determining the reasonableness of the many demands made upon the workers, and which would assuredly be better understood when the knowledge had been gained by personal experience.

### THE STAFF.

The staff numbers seventy-six, and includes twelve residents, who are required to assist with and supervise the work of the outdoor workers. These maids are skilled in the branch of work they are required to undertake, and are appointed from other laundries (preferably commercial), and in some cases are promoted from the outdoor staff, when they have gained sufficient knowledge and have proved themselves capable of supervising the work of others. There can be no doubt that constant skilled supervision is of the utmost importance in maintaining a high standard of work, and in keeping a good tone throughout.

The out-workers, in most cases, are girls who have had experience elsewhere, or girls from school, at 14 years of age, and they are required to obtain a certificate of fitness from the doctor appointed under the Factory Act. The inexperienced girl receives 3s. weekly to commence, and all workers have their food provided. Most institutions supply the food, and no doubt this is fairer to the workers, and therefore better for the authorities in the long run. The system in factories of employing girls who have to provide their own food, out of scanty wages, is not to be commended, and it is well known that the girls buy what they fancy. This generally takes the form of pickles, biscuits, or apples, and ice-cream, food certainly not nourishing for the growing girl who has to work hard day by day. The little girls are very promising, and at the London Hospital they are taught to take some responsibility from the beginning. They generally become good maids, either continuing in the service of the hospital or leaving to take responsible appointments elsewhere.

### Stores and Materials.

The stores, materials of all kinds, and "dressings" for machinery, are ordered by the sister-in-charge, subject to the matron's approval, so that new preparations, if they tend to promote economy, can be utilised. In some cases it will be found that even the head laundress, or superintendent, does not know the price of the soap she is using nor the name of the firm which supplies it! Is it possible for workers to take an interest in the economical use of materials, and in all other points relating to economy, unless the responsible "head" is able to teach them the value of it? All maids and workers should be taught and encouraged to remember the price of nearly everything they use in their work, and to take an interest in saving wherever it is possible. The British race is wasteful and extravagant, and as the price of everything is increasing it is most essential that a close check should be kept on the use of material, the cost of which makes a great demand on the hospital funds. The soap, alkali, starch, etc., is weighed up for each day's use, and an account kept of the quantity used per week.

The London Hospital system undoubtedly supplies a great want in the standard of work in institution laundries, and the method adopted by many in employing women who know nothing about the work, or from other departments at odd times, is one to be condemned. Such a method will only find the workers lacking in interest and enthusiasm, and cause constant perplexity and disappointment to those responsible. Perhaps for this reason alone hospitals have gained the reputation of doing their work in the laundry in a rough way. There can be no reasonable excuse for returning linen badly washed just because it is an institution.

The laundry department is generally very well equipped, and in many cases bettter able to gauge the quantity of linen required to be returned in a given time than a trade laundry, where everything is done for profit; therefore, if the workers are trained in their various duties they ought to be able to do the work better. The London Hospital course of three months' laundry training, with board and residence, at the reasonable fee of 13 guineas, offers exceptional advantages to ladies desirous of learning this branch of work, and to nurses who have finished their training, and wish to gain further experience in hospital management. The pupils are generally successful in obtaining good posts, and those who have shown ability for the work are doing much to improve the standard in other laundries. Notwithstanding the many disappointments, the authorities find much to encourage them in helping working girls to become useful citizens. They need "something to do, something to love, and something to hope for."

*London Hospital, Whitechapel, Washing Return,* 1911.

| Wages of Laundry Hands: | £ | *s.* | *d.* | £ | *s.* | *d.* | £ | *s.* | *d.* |
|---|---|---|---|---|---|---|---|---|---|
| Female ... ... ... ... | £1,899 | 5 | 3 | | | | | | |
| Male ... ... ... ... | 152 | 0 | 6 | 2,051 | 5 | 9 | | | |
| Plus Board ... ... ... | — | | | 800 | 9 | 6 | 2,851 | 15 | 3 |
| Proportion of wages, engineer, stoker, etc. ... ... | 52 | 3 | 11 | | | | | | |
| Board ... ... ... ... | — | | | — | | | 52 | 3 | 11 |
| Materials and sundries: | | | | | | | | | |
| Uniforms ... ... ... | 26 | 9 | 9 | | | | | | |
| Hardware, crockery and brushes ... ... ... | 6 | 1 | 4 | | | | | | |
| Cleaning and chandlery ... | 389 | 13 | 7 | | | | | | |
| Bedding and linen ... ... | 64 | 7 | 0 | | | | | | |
| Printing and stationery ... | 12 | 9 | 11 | | | | | | |
| Drugs, chemicals, disinfectants ... ... ... | 10 | 8 | 0 | | | | | | |
| Dressings and bandages ... | 0 | 5 | 4 | | | | | | |
| Sundries, surgery and dispensary ... ... ... | 0 | 0 | 9 | | | | | | |
| Renewal of furniture ... ... | 1 | 6 | 9 | | | | | | |
| Domestic sundries ... ... | 0 | 14 | 3 | — | | | 511 | 16 | 8 |
| Carriage ... ... ... | — | | | — | | | — | | |
| Fuel Power, and Lighting: | | | | | | | | | |
| Electric current ... ... | 123 | 3 | 10 | | | | | | |
| Gas ... ... ... ... | 37 | 18 | 5 | | | | | | |
| Coal and coke ... ... ... | 684 | 2 | 5 | | | | | | |
| Oil and wood, etc. ... ... | 1 | 9 | 11 | — | | | 846 | 14 | 7 |
| Water ... ... ... ... | — | | | — | | | 17 | 6 | 9 |
| Repairs to Laundry Buildings: | | | | | | | | | |
| Wages ... ... ... ... | 42 | 17 | 11 | | | | | | |
| Materials ... ... ... | 60 | 3 | 5 | | | | | | |
| Annual cleaning ... ... | 68 | 15 | 8 | — | | | 171 | 17 | 0 |
| Repairs to machinery and plant ... ... ... ... | 181 | 18 | 6 | | | | | | |
| Insurance ... ... ... | 0 | 8 | 6 | — | | | 182 | 7 | 0 |
| Insurance (fire and boiler) ... | — | | | — | | | 12 | 2 | 3 |
| Depreciation: | | | | | | | | | |
| Buildings—1½ per cent. on value of buildings, Jan. 1, 1911, £17,256 8s. 9d. (leaves value Jan. 1, 1912, £16,997 11s. 10d.) ... | — | | | 258 | 16 | 11 | | | |
| Machinery—5 per cent. on value at end of 1911, being value Jan. 1, 1911 (£3,746 1s. 1d.) plus £253 8s. 7d. (*i.e.*, renewals and new machinery in 1911) ... ... ... | — | | | 199 | 19 | 6 | | | |
| | | | | | | | 458 | 16 | 5 |
| Interest on original capital, £24,643 15s. ... ... | — | | | — | | | 985 | 15 | 2 |
| Rent ... ... ... ... | — | | | — | | | — | | |
| Rates ... ... ... ... | — | | | — | | | 143 | 1 | 8 |
| Total cost of washing done ... .. ... ... | | | | | | | £6,233 | 16 | 8 |

Total pieces washed, 2,796,795. (Staff, 1,323,977; hospital, 1,472,818. Cost per 100 pieces 4s. 5½d. Weekly average, 53,637.

